# A Preliminary Study on Antimicrobial Susceptibility of *Staphylococcus* spp. and *Enterococcus* spp. Grown on Mannitol Salt Agar in European Wild Boar (*Sus scrofa*) Hunted in Campania Region—Italy

**DOI:** 10.3390/ani12010085

**Published:** 2021-12-31

**Authors:** Francesca Paola Nocera, Gianmarco Ferrara, Emanuela Scandura, Monica Ambrosio, Filomena Fiorito, Luisa De Martino

**Affiliations:** 1 Department of Veterinary Medicine and Animal Production, University of Naples Federico II, Via F. Delpino 1, 80137 Naples, Italy; gianmarco.ferrara@unina.it (G.F.); e.scandura@studenti.unina.it (E.S.); monica.ambrosio@unina.it (M.A.); filomena.fiorito@unina.it (F.F.); luisa.demartino@unina.it (L.D.M.); 2 Task Force on Microbiome Studies, University of Naples Federico II, 80137 Naples, Italy

**Keywords:** *Staphylococcus* spp., *Enterococcus* spp., antimicrobial resistance, wild boars

## Abstract

**Simple Summary:**

During the last decade, an increase in the European wild boar (*Sus scrofa*) population occurred; thus, over the years, wild boars have become an important potential carrier of pathogenic bacteria for both livestock animals and pets, but also for humans. Since antibiotic resistance has become one of the greatest challenges of global public health, the aim of the present study was to define the prevalence and the antibiotic resistance profiles of bacteria grown on the selective medium mannitol salt agar (MSA), isolated from nasal swabs of wild boars hunted in Campania Region (southern Italy). The most prevalent isolated bacteria were represented by the *Staphylococcus* spp. and *Enterococcus* spp. strains, which showed worrying antibiotic-resistant profiles. Consequently, constant surveillance of wild boars is strongly recommended, in order to assess their role as reservoirs of antibiotic resistant bacteria and as sentinels of a possible environmental contamination.

**Abstract:**

The importance of wild boar lies in its role as a bioindicator for the control of numerous zoonotic and non-zoonotic diseases, including antibiotic resistance. Mannitol Salt Agar (MSA) is a selective medium used for isolation, enumeration, and differentiation of pathogenic staphylococci. Other genera such as *Enterococcus* spp. are also salt tolerant and able to grow on MSA. The present study focused on the identification, by matrix assisted laser desorption/ionization-time of flight mass spectrometry (MALDI-TOF-MS), of bacteria grown on MSA isolated from the nasal cavities of 50 healthy wild boars hunted in Campania Region (southern Italy) in the year 2019. In addition, the antimicrobial resistance phenotype of the isolated strains was determined by disk diffusion method. Among genus *Staphylococcus*, coagulase-negative *Staphylococcus* (CoNS) were the most common isolated species, with *Staphylococcus xylosus* as the most prevalent species (33.3%). Furthermore, *Enterococcus* spp. strains were isolated, and *Enterococcus faecalis* was the species showing the highest frequency of isolation (93.8%). For staphylococci, high levels of resistance to oxacillin (93.3%) were recorded. Differently, they exhibited low frequencies of resistance to tested non-β-lactams antibiotics. Among enterococci, the highest resistances were observed for penicillin (93.7%), followed by ampicillin (75%), and ciprofloxacin (68.7%). Interestingly, 43.7% of the isolated strains were vancomycin-resistant. In conclusion, this study reports the phenotypic antibiotic resistance profiles of *Staphylococcus* spp. and *Enterococcus* spp. strains isolated from nasal cavities of wild boars hunted in Campania Region, highlighting that these wild animals are carriers of antibiotic resistant bacteria.

## 1. Introduction

The recent focus on veterinary public health aspects in a One Health framework consisting of game management, with particular reference to wild boars, as reservoirs of important zoonotic diseases, is of great interest for animal health. Wild boar (*Sus scrofa*) is susceptible to numerous viral, bacterial, and parasitic diseases that can have considerable direct and indirect interest for humans, other populations of wild animals, domestic animals, the species itself and the environment [1]. Furthermore, wild boars are often carrier of antibiotic-resistant bacteria, favoring their circulation in human, livestock, and natural environments [2]. Particularly, the circulation of antibiotic-resistant staphylococci in natural ecosystems represents a relevant concern, since these microorganisms can act as vectors of relevant antimicrobial resistance mechanisms and find a natural reservoir in wild boar [3]. Members of the genus *Staphylococcus* are common colonizers of the skin in mammals [4] and due to their ability to coagulate rabbit plasma, staphylococci have been grouped into coagulase-positive staphylococci (CoPS) or coagulase-negative staphylococci (CoNS). Among CoPS, *Staphylococcus aureus* (*S. aureus*) represents the main causative agent of infections such as superficial skin and soft tissue infections, osteomyelitis, and septicemia both in humans and animals [5]. Its medical importance is mainly represented by the emergence and spread of methicillin-resistant *S. aureus* (MRSA), which, for its ability to adapt rapidly to the selective pressure of antibiotics, often presents worrying multidrug resistance profiles. The distribution of multidrug-resistant MRSA among several apparently healthy animal species represents a potential worrying public health issue [6]. Domestic swine are frequently colonized by *S. aureus*, and they are recognized as a main reservoir for MRSA, but studies on MRSA in wild boars are currently scarce, precise MRSA has been identified only in wild boars from Germany and Spain [7,8]. Despite, CoNS have less virulence factors than *S. aureus*, they have become important nosocomial pathogens, and many species colonize the skin and mucous membranes of both humans and animals [9]. Moreover, in wildlife animals, the isolation of CoNS has been reported in recent years. Indeed, Mama et al. [10] reported for the first time the isolation of CoNS in Spain with a percentage of 36.6% from the nose of wild boars, with a high prevalence of species such *Staphylococcus sciuri* (*S. sciuri*) (64/161), *Staphylococcus xylosus* (*S. xylosus*) (21/161), and *Staphylococcus chromogenes* (*S. chromogenes*) (17/161). Furthermore, the isolated species presented antibiotic resistance profiles, with 22.4% showing resistance to at least one antibiotic, and carrying relevant antibiotic resistant genes such as *mec*A and *tet*K genes [10]. Multidrug- and methicillin-resistant *S. sciuri* strains were isolated from wild Ungulates in Spain [11], highlighting the role of these animals as reservoirs of multi-resistant methicillin-resistant *Staphylococcus* strains. So, according to literature, the spread of methicillin and multidrug-resistant CoNS has increased over the years, becoming a worrying threat both for human and veterinary medicine [12,13,14,15,16]. In this scenario, CoNS of both human and veterinary origin represent a great concern, since it is well known their role as reservoirs of antibiotic resistance genes for other pathogenic bacteria such as *S. aureus*, increasing their virulence potential and making them therapeutic challenges [9].

Mannitol salt agar (MSA) plates are generally used for *Staphylococcus* spp. strains isolation [17], but its high concentration of salt (NaCl) is also tolerated by *Enterococcus* spp. and *Micrococcaceae* [18,19,20]. Quiloan et al. [21] reported that *Enterococcus faecalis* (*E. faecalis*) was a mannitol positive strain producing, thus, yellow colonies on MSA; differently from *Enterococcus faecium* (*E. faecium*), which lacked this phenotype.

Bacteria of the genus *Enterococcus*, which are considered harmless commensal in healthy subjects, are often resistant to a number of clinically important antibiotics, and therefore are hired as sentinel microorganisms for tracking trends in resistance to antimicrobials with Gram-positive activity [22]. Enterococci, lactic acid bacteria (LAB), comprise both pathogenic and commensal microorganisms ubiquitous in environment, in fact they can be detected from soil, water, plants, wild animals, birds, and insects, even as gut symbionts. Mostly two subspecies are of particular relevance: *E. faecalis* and *E. faecium*. Clinical isolates of *E*. *faecalis* and *E. faecium* have resistance to many commonly used antimicrobial agents, such as ampicillin and vancomycin [23].

Moreover, enterococci seem to be less virulent in nature than *S. aureus* or Gram-negative bacteria, but vancomycin-resistant *Enterococcus* (VRE) can cause a variety of infections and represents a pathogen of growing concern; in fact, in recent years, an increase in invasive VRE human infections has been reported worldwide [24]. However, like MRSA, VRE is endemic in hospital settings [25] and it is becoming difficult to manage, even though VRE is one of the first documented antibiotic resistant bacteria with primary origin in animal farming [26].

Different bacterial populations are present within the nasal cavities of wild boars. The aim of this study was to assess if wild boars could represent a potential risk for other animals, humans, and the environment, as a source of multidrug-resistant strains able to grow on MSA medium.

## 2. Materials and Methods

### 2.1. Ethical Approval

This study did not involve the use of living wild boars, thus ethical approval was not required. All nasal swabs were collected from wild boars during the hunting season by licensed and specialized hunters. Furthermore, before the opening of the hunting season of the year 2019, hunters trained for a proper sampling of wild boar nasal cavities. After sampling, swabs were held at 4 °C during the transport to the Microbiology laboratory.

### 2.2. Sample Collection

Nasal swabs from apparently healthy 50 wild boars were collected at the time of capture in the hunting season. Samples were collected from both male and female wild boars, weighing between 20 kg and 100 kg. The specific wild boar hunting area was the one located in the province of Salerno in Campania Region (southern Italy). The hunt was conducted by authorized hunter teams during the normal hunting period (from 1 October 2019 to 31 December 2019). The sampling was performed by inserting and rotating a single swab per animal in both nostrils. Each swab was then placed in Stuart W/O CH (Aptaca Spa, Asti, Italy) transport medium and transferred within 48 h, by using an icebox, to the laboratory for bacteriological examination.

### 2.3. Bacterial Isolation and Identification

Nasal swab samples were streaked onto mannitol salt agar (MSA) (Liofilchem Srl, Teramo, Italy) and incubated aerobically at 37 °C for 24 h, in order to collect bacterial isolates able to grow on this medium. Colonies were firstly identified by standard, rapid screening techniques: colony morphology, presence, or absence of mannitol fermentation on MSA, Gram-staining, catalase reaction, and for staphylococci also the tube coagulase reaction (Oxoid, Ltd., Hampshire, UK) was performed.

Single colonies were subcultured on Columbia Sheep Blood agar (Liofilchem Srl, Teramo, Italy) and after incubation at 37 °C for 24 h, the isolates were identified using the matrix assisted laser desorption/ionization-time of flight mass spectrometry (MALDI-TOF-MS) (Bruker Daltonics Inc., Bremen, Germany).

Only staphylococci and enterococci isolates were preserved in 16% *v*/*v* glycerol broth and in Microbank tubes (Pro-Lab Diagnostics, Round Rock, TX, USA) at −80 °C for further investigation.

### 2.4. Antibiotyping of Staphylococcus spp. and Enterococcus spp.

Antibiotic resistance profiles of the recovered isolates were evaluated by disk diffusion method on Mueller Hinton agar plates (Liofilchem, Teramo, Italy). All the isolated staphylococci and enterococci were tested for their susceptibility to the following antibiotics: amoxicillin-clavulanate (AUG, 20/10 µg), ampicillin (AMP, 10 µg), ciprofloxacin (CIP, 5 µg), erythromycin (E, 15 µg), gentamicin (CN, 10 µg), imipenem (IMI, 10 µg), penicillin (P, 10 IU), sulfamethoxazole-trimethoprim (SXT, 1.25/23.75 μg), tetracycline (TE, 30 µg), and vancomycin (VA, 30 µg). Staphylococci were tested also for cefotaxime (CTX, 30 µg), cefoxitin (FOX, 30 µg), cephalothin (KF, 30 µg), clindamycin (CD, 2 µg), enrofloxacin (ENR, 5 µg), and oxacillin (OX, 1 µg). The tested antibiotic belonged to 10 different classes. The antimicrobial susceptibility testing results were interpreted according to the Clinical and Laboratory Standards Institute guidelines [27] and to the European Committee on Antimicrobial Susceptibility Testing [28]. Furthermore, isolates were classified as multidrug-resistant (MDR), extensively drug-resistant (XDR) and pandrug-resistant (PDR) strains according to Magiorakos et al. [29].

## 3. Results

### 3.1. Bacterial Strains Isolated on MSA

During the hunting season of the year 2019, 50 nasal swabs streaked on MSA and microbiologically analyzed. Precisely, 37 (74%) swabs yielded Gram-positive isolates, while 13 (26%) nasal swabs did not yield any Gram-positive bacteria growth. From all positive processed specimens, pure cultures were detected in 32 nasal swabs while in 5 of them, the co-presence of two different bacterial species was detected. Altogether a total of 42 bacterial strains was isolated. Specifically, 38 (90.5%) of the isolated strains resulted to be fermenting mannitol, by forming yellow colonies on MSA, whilst 4 (9.5%) strains were mannitol-negative by producing light pink colonies. Catalase test, which is essential to differentiate *Staphylococcus* spp. (catalase positive) from *Enterococcus* spp. (catalase negative), gave positive results for 26 bacterial strains, the remaining 16 isolates turned out to be negative instead. All the recovered strains were negative to oxidase test. Furthermore, for the 15 Gram-positive cocci seen in tetrads and clusters on the smear, the performed staphylocoagulase tests resulted to be all negative, pointing out the absence of coagulase-positive staphylococci like *S. aureus*. All the above-mentioned results are reported in Table 1.

### 3.2. Identification of Bacterial Species Recovered from Wild Boars

**Figure 1:**
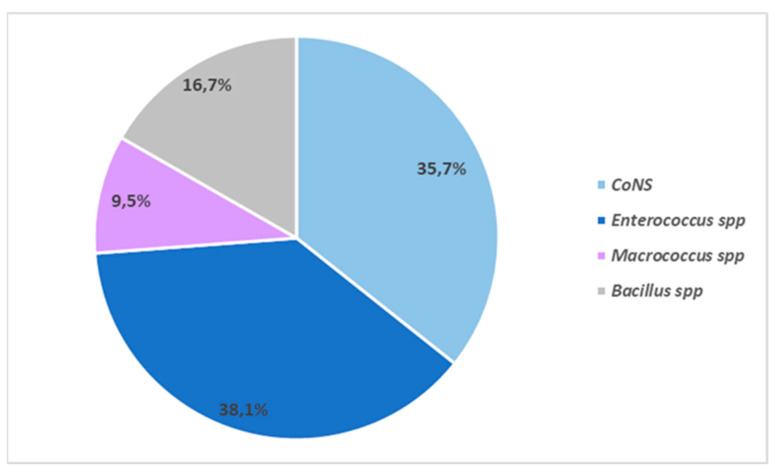
Frequency of isolation of Gram-positive bacteria grown on MSA.

From a total of 42 Gram-positive isolates, 15 belonged to *Staphylococcus* spp. (35.7%) and were all CoNS, whereas 16 belonged to *Enterococcus* spp. (38.1%) (Figure 1). However, on MSA, we detected the growth of other Gram-positive bacteria such as *Macrococcus* spp. (9.5%; 4/42 strains) and *Bacillus* spp. (16.7%; 7/42 strains), which were identified with lower frequency values as represented in Figure 1.

Among isolated CoNS, five different species were identified in total, as reported in Table 2. *S. xylosus* resulted to be the most prevalent species (33.3%; 5/15 strains), followed by *S. chromogenes* (26.7%; 4/15 strains), *Staphylococcus hyicus* (*S. hyicus*) (20%; 3/15 strains), *S. sciuri* (13.3%; 2/15 strains), and *Staphylococcus simulans* (*S. simulans*)(6.7%; 1/15 strains) (Table 2); whereas among enterococci, *E. faecalis*, known for its wide tolerance to high concentrations of salt and able to ferment mannitol, was the most predominant species (93.8%; 15/16 strains). The other enterococcal isolate was represented by *Enterococcus casseliflavus* (*E. casseliflavus*) (6.2%; 1/16 strains) showing the same phenotype on MSA.

Furthermore, from five wild boar nasal samples two different bacterial species were recovered. Precisely, the following co-present species were found per animal (sample ID): Bacillus megaterium/*E. faecalis* (39a/39b); *S. sciuri*/*S. xylosus* (40a/40b); *S. chromogenes*/Bacillus licheniformis (45a/45b); *S. hyicus*/*S. chromogenes* (47a/47b), while from the nasal swab of one animal, two isolates with different colony morphology were identified as *E. faecalis* (31a/31b).

All the strains isolated on MSA were identified by MALDI-TOF-MS with good (log) scores (1.9 ≤ x ≥ 2.0) (Table S1 in Supplementary Data).

### 3.3. Phenotypic Antibiotic Resistant Profiles of Staphylococcus spp. and Enterococcus spp. Isolates

In this study, the antibiotic resistance profiles of CoNS and *Enterococcus* spp. strains were evaluated. The antibiotic resistance profiles of *Macrococcus* spp. and *Bacillus* spp. isolates were not analyzed, due to the low number of the collected isolates.

**Figure 2:**
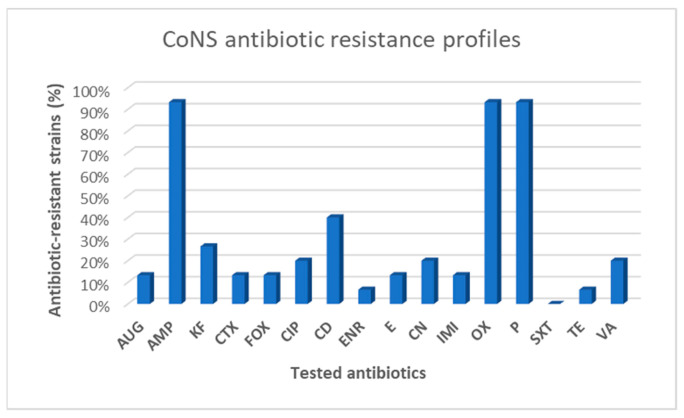
Antimicrobial resistance profiles of CoNS isolated from wild boars. Antibiotics: AUG: amoxicillin-clavulanate; AMP: ampicillin; KF: cephalothin; CTX: cefotaxime; FOX: cefoxitin; CIP: ciprofloxacin; ENR: enrofloxacin; E: erythromycin; CN: gentamicin; IMI: imipenem; OX: oxacillin; P: penicillin; SXT: sulfamethoxazole-trimethoprim; TE: tetracycline; VA: vancomycin.

Both CoNS and *Enterococcus* spp. strains showed relevant resistance frequencies to the selected antibiotics. As reported in Figure 2, for CoNS interesting levels of resistance were observed for β-lactam antibiotics. Particularly, the highest levels of resistance were recorded for oxacillin together with penicillin and ampicillin with 14/15 strains resistant to these antibiotics (93.3%). Differently, high susceptibility levels were detected for cefoxitin (86.7%; 13/15 strains). Surprisingly, CoNS exhibited low frequencies of resistance to tested non-β-lactams antibiotics with values never higher than 20% for ciprofloxacin, erythromycin, enrofloxacin, gentamicin, and tetracycline, whereas a slightly higher level of resistance was recorded for clindamycin (40%; 6/15 strains). It is worth noting that resistance to vancomycin was detected in three CoNS strains (20%). No resistance was recorded for the antibiotic sulfamethoxazole-trimethoprim (Figure 2). One isolate, identified as *S. hyicus* (ID 49), resulted susceptible to all tested antibiotics (Table 2).

According to the classification given by Magiorakos et al. [29], 40% (6/15) of CoNs strains (ID 18, 20, 23, 34, 36, 47b) MDR strains were detected. Moreover, the isolate S. xylosus (ID 22) was identified as XDR, showing resistance to at least one antimicrobial agent of each tested antibiotic classes. No PDR CoNS were isolated. CoNS antibiotic resistance profiles, and the categorization in MDR and XDR strains are summarized in Table 2.

**Table 2:** Antibiotic resistance phenotype of isolated CoNS strains.

ID	CoNs Identified Species	Antibiotic Resistance Profiles	MDR	XDR
2	*S. sciuri*	AMP, CIP, OX, P		
18	*S. chromogenes*	AMP, KF, CD, E, OX, P, VA	X **	
20	*S. chromogenes*	AMP, CD, CN, OX, P	X	
22	*S. xylosus*	AUG, AMP, KF, CTX, FOX, CIP, CD, ENR, E, CN, IMI, OX, P, TE, VA		X
23	*S. xylosus*	AMP, KF, OX, P	X	
34	*S. simulans*	AMP, KF, CD, OX, P, VA	X	
36	*S. xylosus*	AUG, AMP, CTX, FOX, CD, IMI, OX, P	X	
40a	*S. sciuri*	AMP, OX, P		
40b	*S. xylosus*	AMP, OX, P		
44	*S.hyicus*	AMP, OX, P		
45a	*S. chromogenes*	AMP, OX, P		
47a	*S. hyicus*	AMP, OX, P		
47b	*S. chromogenes*	AMP, CD, CN, OX, P	X	
49	*S. hyicus*	----- *		

* Sample ID 49 is susceptible to all tested antibiotics. ** X: presence of MDR or XDR phenotype. Antibiotics: AUG: amoxicillin-clavulanate; AMP: ampicillin; KF: cepalothin; CTX: cefotaxime; FOX: cefoxitin; CIP: ciprofloxacin; ENR: enrofloxacin; E: erythromycin; CN: gentamicin; IMI: imipenem; OX: oxacillin; P: penicillin; SXT: sulfamethoxazole-trimethoprim; TE: tetracycline; VA: vancomycin.

**Figure 3:**
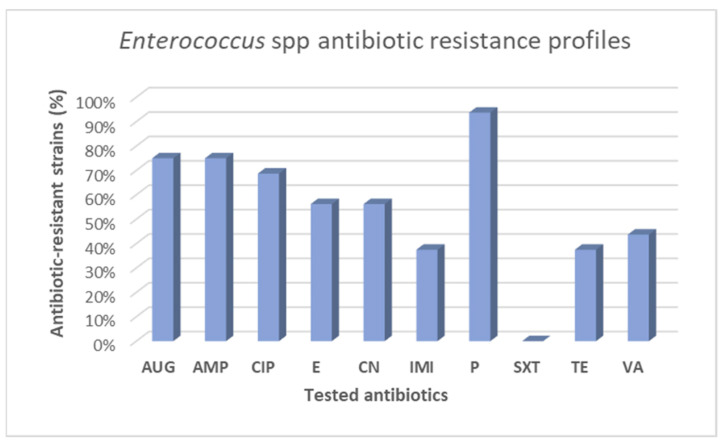
Antibiotic resistance profiles of *Enterococcus* spp. strains isolate from wild boars. Antibiotics: AUG: amoxicillin-clavulanate; AMP: ampicillin; CIP: ciprofloxacin; E: erythromycin; CN: gentamicin; IMI: imipenem; P: penicillin; SXT: sulfamethoxazole-trimethoprim; TE: tetracy-cline; VA: vancomycin.

The antibiotic resistance profiles of the isolated enterococci are exposed in Figure 3. Resistance to penicillin was found in almost all of the analyzed samples (93.7%; 15/16 strains). It should be also noted that more than half of the isolates were also resistant to ampicillin and amoxicillin-clavulanic acid, which presented the same frequency of resistance (75%; 12/16 strains), followed by ciprofloxacin (68.7%) (Figure 3). Interesting levels of resistance were observed also for gentamicin and erythromycin equally (56.2%; 9/16 strains), whilst lower values of resistance were recorded for tetracycline and imipenem with 6/16 resistant strains (37.5%). Resistance to vancomycin was detected, in 7 of 16 total isolates (43.7%) as represented in Figure 3. Intriguingly, from a wild boar nasal swab two isolated of the same species *E. faecalis* were recovered, showing a different antimicrobial resistance phenotype. Precisely, ID 31b-*E. faecalis* isolate appeared to be a MDR strain, the other *E. faecalis* (ID 31a) strain resulted to be more susceptible, with only two phenotypic resistances as reported in Table 3. As for CoNS, also for enterococci no resistance against sulfamethoxazole-trimethoprim was observed. However, the overall prevalence of antibiotic resistance among *Enterococcus* spp. isolates evaluated in the present study emerged to be high; 62.5% of isolated enterococci (10/16) were MDR strains, while 25% (4/16) were identified as XDR isolates. Pandrug resistance was not detected. Antibiotic resistance profiles of isolated enterococci, and their categorization in MDR and XDR strains are indicated in Table 3.

## 4. Discussion

During the last decade, an increasing of the European wild boar population has occurred; thus, over the years wild boars have become an important potential source of pathogenic bacteria for both livestock animals and pets, especially hunting dogs, but also for humans, due to a major consumption of wild boar meat linked to growing hunting activities [1]. In Italy, there are many reports on the reservoir role played by wild boars for zoonotic bacteria such as *Brucella* spp., *Mycobacterium* spp., and *Salmonella* spp., which often show worrying antibiotic resistance profiles [30,31,32,33,34]. Antibiotic resistance is currently one of the greatest challenges of global public health. Globally, a continued increase in the number of resistant bacteria is constantly recorded, in both humans and animals [35]. This scenario is generally linked to an improper use of antibiotics in veterinary and human clinical settings, which results in an environmental contamination. Consequently, wildlife animals, particularly wild ungulates, and wild birds, are becoming reservoirs and spreaders of multidrug-resistant bacteria [10,11,14], and it is cause of great concern, since these free-living animals are not subjected to antibiotic pressure. However, information on antibiotic resistant profiles of opportunistic pathogens such as staphylococci and enterococci isolated from wild boars are still scarce both in Italy and worldwide. To the best of our knowledge, this is the first study in Italy on the prevalence and antibiotic resistance phenotype of *Staphylococcus* spp. and *Enterococcus* spp. recovered from nasal samples of wild boars in southern Italy. All 50 nasal swabs, collected in the hunting season 2019, were streaked on MSA. Although MSA is a selective and differential medium generally used for staphylococci isolation, in the present study, not only the growth of CoNS, but also of *E. faecalis* and *E. casseliflavus* strains was detected. This latter obtained result agrees with Quiloan et al. [21], who already reported enterococci ability to grow on MSA, since they are salt tolerant. Furthermore, in this study the isolation of some strains of *Macrococcus* spp., genus closely related to genus *Staphylococcus* consisting of eleven species typically found as commensals in a range of animal hosts [36], and *Bacillus* spp. were observed, but with a lower prevalence than CoNS and enterococci. It is worth of noting that almost all the isolated strains resulted to be mannitol positive (90.5%; 38/42 strains); the remaining four strains were not able to ferment mannitol (9.5%). Specifically, all *E. faecalis* strains but also *E. casseliflavus* isolate fermented mannitol on MSA, according to the data described by Quiloan et al. [21]. Referring to CoNS, 12 isolated were mannitol fermenting, whilst a strain of each of the following species: *S. chromogenes*, *S. hyicus*, and *S. simulans* grew by forming light pink colonies on MSA. According to literature, CoNS ability to ferment mannitol on MSA vary among different species and strains of the same species [37,38,39]. The percentage of CoNS was of 35,8% and it is not surprising since CoNS are normal inhabitants of skin and mucosal membranes of animals and humans. Moreover, our positivity percentage is similar to the one recorded by Mama et al. [10], which reported a prevalence value of 37.7% for CoNS isolated from nasal swabs of wild boars hunted in Spain in 2016. Differently from Mama et al. [10], herein no coagulase positive staphylococci (CoPS) were isolated, even though wild boar is considered one of the main *S. aureus* reservoirs among wild animals [40,41]. Despite CoNS have long been considered less pathogenic than *S. aureus* and other CoPS, CoNS infections have become increasingly difficult to treat for the increased number of circulating multidrug-resistant strains in recent years [42]. In this study, CoNS displayed interesting resistance profiles. Particularly, CoNS showed the highest levels of resistance to oxacillin together with penicillin and ampicillin with 14/15 strains resistant equally to these antibiotics (93.3%), whilst low levels of resistance were recorded for cefoxitin (13.3%). Interestingly, low frequencies of resistance (<50%) were observed for the tested non-β-lactams antibiotics, such as ciprofloxacin, clindamycin erythromycin, enrofloxacin, gentamicin, and tetracycline. Forty percent of the isolated CoNS resulted to be multidrug-resistant and an extensively drug resistance profiles of observed only in a strain of *S. xylosus*. This result is of great interest, since CoNS are known to be reservoirs of resistance genes; that can easily be transmitted to other more virulent staphylococci like *S. aureus* and *Staphylococcus pseudintermedius* (*S. pseudintermedius*), decreasing the chances of success of therapeutic treatments [9]. Furthermore, the prevalence of MDR CoNS strains found here is not only in accordance with data reported for CoNS isolated from wild boars of other European countries [10,11], but also in CoNS strains isolated from pets suffering from skin infections [16].

Referring to enterococci, the prevalence of isolation from the collected nasal swabs was of 38%. This is an intriguing result, since it is generally unusual recover enterococci from nasal samples of animals, which are reported here for the first time in wild boars. Enterococci are known to be part of the gastrointestinal flora of humans and animals, but being also opportunistic pathogens, they have been recognized, particularly *E. faecalis* and *E. faecium*, as a significant cause of nosocomial infections [43]. *E. faecalis* was the most predominant enterococcal species (93.8%; 15/16 strains) isolated from the here analyzed wild boar nasal swabs. This obtained interesting result can be linked to wild boar rooting activity and to the high presence of *E. faecalis* in the environment, since it is considered a fecal indicator organism, which can be found and transmitted by different environmental matrices (e.g., water, plants, soil, sediments, and sand). Thus, wildlife animals such as wild boars, birds, and deer can be sources of enterococci in urban and rural environments, either through direct deposition or in runoff [43]. In addition, the isolated *Enterococcus* spp. strains displayed relevant resistance profiles. Resistance to penicillin was found in almost all of the analyzed samples (93.7%) and 75% of the isolates were also resistant to ampicillin and amoxicillin-clavulanic acid, followed by ciprofloxacin (68.7%). Similar resistance profiles were described also by Olivera de Araujo et al. [44] for *E. faecalis* and other *Enterococcus* spp. strains isolated from fecal samples of wild Pampas foxes and Geoffroy’s cats in the Brazilian Pampa biome. Furthermore, 43.7% of the isolated strains all identified as *E. faecalis* were resistant to vancomycin. As reported by Zaheer et al. [45] which studied the antibiotypes of *E. faecalis* isolated from humans, cattle, and environmental sources with a One Health approach, also our VRE *E. faecalis* were multi-resistant to β-lactams, macrolides, aminoglycosides, fluoroquinolones, and tetracyclines. The most remarkable result emerging in this study is that MDR and XDR strains isolated from nasal wild boar swabs were 62.5% and 25%, respectively. Indeed, it is known that enterococci can acquire resistance to numerous antimicrobial agents, acting also as efficient donor of resistance genes.

The findings of the present study highlighted the isolation of CoNS and *Enterococcus* strains showing worrying antibiotic resistance profiles in wild boars hunted in Campania Region, southern Italy. The presence of MDR and XDR strains in nasal sample underlines how these animals may contribute to the spread of resistant strains and their transmission to other animals or even to humans. Furthermore, the occurrence of multi-resistance in non-clinical strains may be considered a virulence characteristic that bacteria use to improve their survival, proliferation, and their ability to colonize hosts. Therefore, a continuous monitoring of the occurrence of these opportunistic bacteria in wildlife is desirable. In addition, a constant surveillance of wild boars would be worthwhile, to assess their role as reservoirs of antibiotic resistant bacteria and as sentinels of a possible environmental contamination.

## 5. Conclusions

The present study reveals high colonization and antibiotic resistance in staphylococcal and enterococcal isolates from nasal cavities of wild boars. Furthermore, nasal colonization of multi-resistant CoNS and enterococci in these wild animals is alarming. A positive aspect of these results was the absence of coagulase positive staphylococci, as methicillin-resistant *S. aureus*, but this could be linked to the limitation of this study, represented by the low number of collected samples. So, continuous monitoring and further surveillance studies are required to better understand the emergence and spread of bacterial strains resistant to antibiotics in wild ecosystems. The wild boar represents an important sentinel animal and the bacteria of the two genera, *Staphylococcus* and *Enterococcus*, may be considered relevant bacterial indicators of antibiotic resistance, a global public health concern.

## Figures and Tables

**Table 1:** Phenotypic features of bacteria isolated on MSA from wild boar nasal swabs.

ID	D-Mannitol Fermentation	Catalase Test	Oxidase Test	Staphylocoagulase Test
2	+ *	+	− *	−
7	+	−	−	NA **
9	+	−	−	NA
10	+	−	−	NA
11	+	−	−	NA
12	+	−	−	NA
13	+	−	−	NA
14	+	−	−	NA
17	+	−	−	NA
18	−	+	−	−
19	+	+	−	NA
20	+	+	−	−
21	+	+	−	NA
22	+	+	−	−
23	+	+	−	−
25	+	−	−	NA
27	+	+	−	NA
28	+	+	−	NA
29	+	+	−	NA
30	+	−	−	NA
31a	+	−	−	NA
31b	+	−	−	NA
32	−	+	−	NA
33	+	+	−	NA
34	−	+	−	−
35	+	−	−	NA
36	+	+	−	−
37	+	−	−	NA
38	+	−	−	NA
39a	+	+	−	NA
39b	+	−	−	NA
40a	+	+	−	−
40b	+	+	−	−
43	+	+	−	NA
44	+	+	−	−
45a	+	+	−	−
45b	+	+	−	NA
46	+	−	−	−
47a	+	+	−	NA
47b	+	+	−	−
48	+	+	−	−
49	−	+	−	−

* +: positive; * −: negative; ** NA: not applicable.

**Table 3:** Phenotypic antibiotic resistance profiles of collected *Enterococcus* spp. strains.

ID	*Enterococcus* Species	Antibiotic Resistance Profiles	MDR	XDR
7	*E. faecalis*	AUG, AMP, CIP, IMI, P	X *	
9	*E. faecalis*	AUG, AMP, CIP, E, CN, IMI, P		X
10	*E. faecalis*	AUG, AMP, CIP, E, CN, IMI, P, TE, VA		X
11	*E. faecalis*	AUG, AMP, CIP, E, CN, IMI, P	X	
12	*E. faecalis*	AUG, AMP, E, CN, P	X	
13	*E. casseliflavus*	CIP, IMI, P	X	
14	*E. faecalis*	CIP, IMI, P	X	
17	*E. faecalis*	AUG, AMP, CIP, IMI, P, TE	X	
25	*E. faecalis*	AUG, AMP, CIP, E, CN, P, VA		X
30	*E. faecalis*	AUG, AMP, CIP, E, P, VA	X	
31a	*E. faecalis*	P, TE		
31b	*E. faecalis*	AUG, AMP, CIP, CN, P, TE, VA	X	
35	*E. faecalis*	AUG, AMP, E, P, VA	X	
37	*E. faecalis*	AUG, AMP, CIP, E, CN, P, TE, VA		X
38	*E. faecalis*	E, CN		
39b	*E. faecalis*	AUG, AMP, CN, P, TE, VA	X	

* X: presence of MDR or XDR phenotype. Antibiotics: AUG: amoxicillin-clavulanate; AMP: ampicillin; CIP: ciprofloxacin; E: erythromycin; CN: gentamicin; IMI: imipenem; P: penicillin; SXT: sulfamethoxazole-trimethoprim; TE: tetracycline; VA: vancomycin.

## Supplementary Materials

The following are available online at https://www.mdpi.com/article/10.3390/ani12010085/s1, Table S1: Matrix assisted laser desorption/ionization-time of flight mass spectrometry (MALDI-TOF-MS) identification of Gram-positive bacteria isolated on Mannitol Salt Agar (MSA).
