# Supplementary material for: A Preliminary Study on Antimicrobial Susceptibility of Staphylococcus spp. and Enterococcus spp. Grown on Mannitol Salt Agar in European Wild Boar (Sus scrofa) Hunted in Campania Region—Italy
Source: Animals (Basel). 2021 Dec 31;12(1):85. doi: 10.3390/ani12010085 (PMC8749723; doi:10.3390/ani12010085)
Supplement: Supplementary file 1 [file animals-12-00085-s001.zip › animals-1511461-supplementary.pdf]

Supplementary Materials

Supplementary Table S1: Matrix assisted laser desorption/ionization-time of flight mass spectrometry **(**MALDI-TOF-MS) identification of Gram-positive bacteria isolated on Mannitol Salt Agar (MSA).

ID	Genus and species identification	score value
2	*Staphylococcus sciuri*	1.92
7	*Enterococcus faecalis*	2.50
9	*Enterococcus faecalis*	2.36
10	*Enterococcus faecalis*	2.39
11	*Enterococcus faecalis*	2.36
12	*Enterococcus faecalis*	2.40
13	*Enterococcus casseliflavus*	2.15
14	*Enterococcus faecalis*	2.41
17	*Enterococcus faecalis*	2.47
18	*Staphylococcus chromogenes*	2.24
19	*Macrococcus canis*	2.05
20	*Staphylococcus chromogenes*	2.19
21	*Macrococcus canis*	2.22
22	*Staphylococcus xylosus*	2.03
23	*Staphylococcus xylosus*	2.07
25	*Enterococcus faecalis*	2.36
27	*Macrococcus canis*	2.03
28	*Bacillus mojavensis*	1.91
29	*Bacillus subtilis*	1.92
30	*Enterococcus faecalis*	2.01
31a	*Enterococcus faecalis*	2.31
31b	*Enterococcus faecalis*	2.31
32	*Macrococcus canis*	2.22
33	*Bacillus amyloliquefaciens*	1.85
34	*Staphylococcus simulans*	2.33
35	*Enterococcus faecalis*	2.31
36	*Staphylococcus xylosus*	2.12
37	*Enterococcus faecalis*	2.31
38	*Enterococcus faecalis*	2.32
39a	*Bacillus megaterium*	1.96
39b	*Enterococcus faecalis*	2.32
40a	*Staphylococcus sciuri*	1.95
40b	*Staphylococcus xylosus*	2.01
43	*Bacillus licheniformis*	1.91
44	*Staphylococcus hyicus*	1.91
45a	*Staphylococcus chromogenes*	2.24
45b	*Bacillus licheniformis*	1.90
46	*Bacillus pumilus*	1.71
47a	*Staphylococcus hyicus*	1.90
47b	*Staphylococcus chromogenes*	2.14
48	*Staphylococcus xylosus*	2.20
49	*Staphylococcus hyicus*	1.93
